# MG-63 cells proliferation following various types of mechanical stimulation on cells by auxetic hybrid scaffolds

**DOI:** 10.1186/s40824-016-0079-x

**Published:** 2016-11-07

**Authors:** Hong Jin Choi, Jun Jae Lee, Jung Bok Lee, Hak-Joon Sung, Jung-Woog Shin, Ji Won Shin, Yanru Wu, Jeong Koo Kim

**Affiliations:** 1 Department of Biomedical Engineering, Inje University, Obang-Dong, Gimhae, Gyeongnam 621-749 South Korea; 2 Department of Biomedical Engineering, Vanderbilt University, Nashville, TN 37212 USA

**Keywords:** Negative Poisson’s ratio, PLGA, Hydroxyapatite, Osteoblast, Dynamic compression stimulation

## Abstract

**Background:**

Mechanical properties and cyto-compatibility of a composite scaffold which possessed negative (−) Poisson’s ratio (NPR) was investigated for effective load transfer from auxetic scaffold to cell.

**Methods:**

Organic/inorganic composite scaffolds were prepared by mixing hydroxyapatite (HA) to poly(lactide-co-glycolide) (PLGA). To induce NPR in composite scaffold, 3-directional volumetric compression was applied during the scaffold fabrication at adequate temperature(60°C). The pore size of scaffold ranged between 355–400 μm.

**Results:**

Poisson’s ratios of NPR scaffolds and control scaffolds were −0.07 and 0.16 at 10 % strain. For stable physical stimulating to loaded cells, ceramic/polymer composite scaffold was prepared by incorporating HA in PLGA to increase mechanical strength. Compressive strength of the HA/PLGA composite scaffold (15 wt. % HA to PLGA) was about 21.7 % higher than that of PLGA-only scaffold. The recovery rates of the NPR composite scaffold after applying compression in the dry and wet states were 90 % and 60 %, respectively. Also the composite scaffold was shown to have better hydrophilicity (61.9°) compared to the PLGA-only scaffolds (65.3°). Cell proliferation of osteoblast-like cell line (MG-63) in the composite scaffold was 20 % higher than in PLGA-only scaffold at static compressive stimulation. For dynamic compressive stimulation (15 min cyclic interval), cell proliferation in the composite scaffold was 2 times higher than that of in PLGA-only scaffold. In conclusion, NPR composite (HA/PLGA) scaffold was effective in isotropic compressive load delivery for osteogenic cell proliferation.

**Conclusion:**

This composite scaffold with stimulation can be used as tissue engineered scaffold and dynamic cell culture system for bone tissue regeneration.

## Background

In recent years, biodegradable polymeric scaffold has been widely used for three-dimensional (3-D) cell culture platform to regenerate tissue-based artificial organs [1]. Scaffold fabrication requires consideration of many mechanical factors in order to mimic the native tissue, such as pore size, porosity, and physical property. For example, the most effective pore size of a polymeric scaffold for bone tissue regeneration is known to be 380–405 μm [2–4].

Not only are these mechanical properties of scaffold important for tissue regeneration, but also the physical stimulation applied to cells through the scaffold during culture plays an important role in cell proliferation and have become an important factor in tissue engineering. The tissues are subjected to mechanical stimulation during daily activities in biomimetic environments. Bone tissue modifies its structure by sensing mechanical stresses generated by dynamic loading and unloading cycles in vivo, which, in turn, generates electric current that triggers remodeling activities and bone cell proliferation [5–9]. Fukuda and Yasuda discovered the pieozoelectric theory which showed that bone tissue is transformed by the dynamic load and cycle in vivo. In summary, through mechanical stimulation, the density of bone tissue was improved. Based on this, there are various methods of mechanical stimulation to affect cell behaviors in vitro such as compressive loading, longitudinal stretch, substrate bending, plane substrate distention, and fluid shear systems [8–10].

The Poisson’s ratio is the negative strain ratio of longitudinal strain divided by the transverse strain. If a material has a negative Poisson’s ratio, it can conduct high compressibility in multi axial directions and is referred to as auxetic material [11]. Lakes proposed that application of 3-axis compression and heat treatment above the softening temperature to a negative Poisson’s ratio for auxetic material in order to produce foam [11]. Lakes and Choi formed a re-entrant structure using a 3.5:1 compression ratio to achieve a foam scaffold with a negative Poisson’s ratio using polyurethane [11–13]. PLGA scaffolds are widely used in bone tissue engineering, but few studies have investigated auxetic biodegradable scaffold [14]. A scaffold that has a negative Poisson’s ratio was shown to have isotropic compression around pores when compression was applied to a material in one direction, and it confirmed the proliferation of the bone tissue (MG-63 cell) [14].

In This study, we checked NPR PLGA scaffold and NPR composite scaffold’s various properties and evaluated the relative impact upon cell (MG-63) proliferation. Specifically, the cells in a HA/PLGA scaffold with NPR will receive compressive stimulus from all directions, despite a stimulus only being applied in one direction. Hydroxyapatite (HA) is known inorganic material that is similar to natural bone tissue in terms of its inorganic components, chemical composition and crystallographic characteristics. These advantages of HA in the PLGA scaffold will enhance biocompatibility and mechanical properties of a scaffolds for osteogenic cell growth. Consequently, 3-D scaffolds with a negative Poisson’s ratio would be expected to have a positive effect on bone cell proliferation. Therefore, this study was conducted to fabricate auxetic HA/PLGA and investigate the effects of bone cell proliferation following various dynamic compressive stimuli

## Methods

### Materials

Poly(D,L-lactic-co-glycolic acid) (PLGA) was purchased from Lakeshore Biometerials (Essen, Germany) and Hydroxyapatite (HA) was form Sigma-Aldrich (St. Louis, MO) were used for scaffold fabrication. The molar ratio of lactide to glycolide was 50/50 and their molecular mass averages of the weight (Mw) and number (Mn) are 69 kDa and 42 kDa, respectively. MG-63 osteoblast-like cell for this study was obtained from KCLB (Seoul, Korea) Dulbecco’s Modified Eagle Medium (DMEM) was obtained from Welgene (Daegu, Korea), fetal bovine serum (FBS) was purchased from Gibco (Thermo Fisher Scientific, Waltham, USA)and penicillin/streptomycin (P/S) was purchased from Sigma-Aldrich (St. Louis, MO)

### Fabrication of scaffolds

HA/PLGA composite scaffold was fabricated using a solvent casting/salt leaching method. Briefly, PLGA was dissolved in chloroform at a concentration of 10 w/v % and then HA was added at different concentration of 0, 5, 10, and 15 wt. % to PLGA. The HA/PLGA composite solution was mixed well with two different sodium chloride particles (355–400 μm and 500–600 μm) to generate pore structures. The mixture was then poured into a Teflon mold (1.5 × 1.5 × 1.5 cm3 for control group and 2 × 2 × 2 cm3 for experimental group) and dried at room temperature for 24 h. The dried samples were immersed in distilled water for 2 days to remove salt particles. Finally, specimens were freeze-dried for 24 h to remove distilled water [15–17].

To induce NPR, the 3-axis permanent volumetric compression with heat treatment technique was proposed [11]. Briefly, porous scaffolds were annealed at 60 °C in heating oven under the three orthogonal directional compression for 10 min. The compression ratio was 2.37:1 (2 × 2 × 2 cm3 to 1.5 × 1.5 × 1.5 cm3). After treatment, the specimens were allowed to cool down at room temperature for 24 h.

### Measurement of Poisson’s ratio

**Fig. 1:**
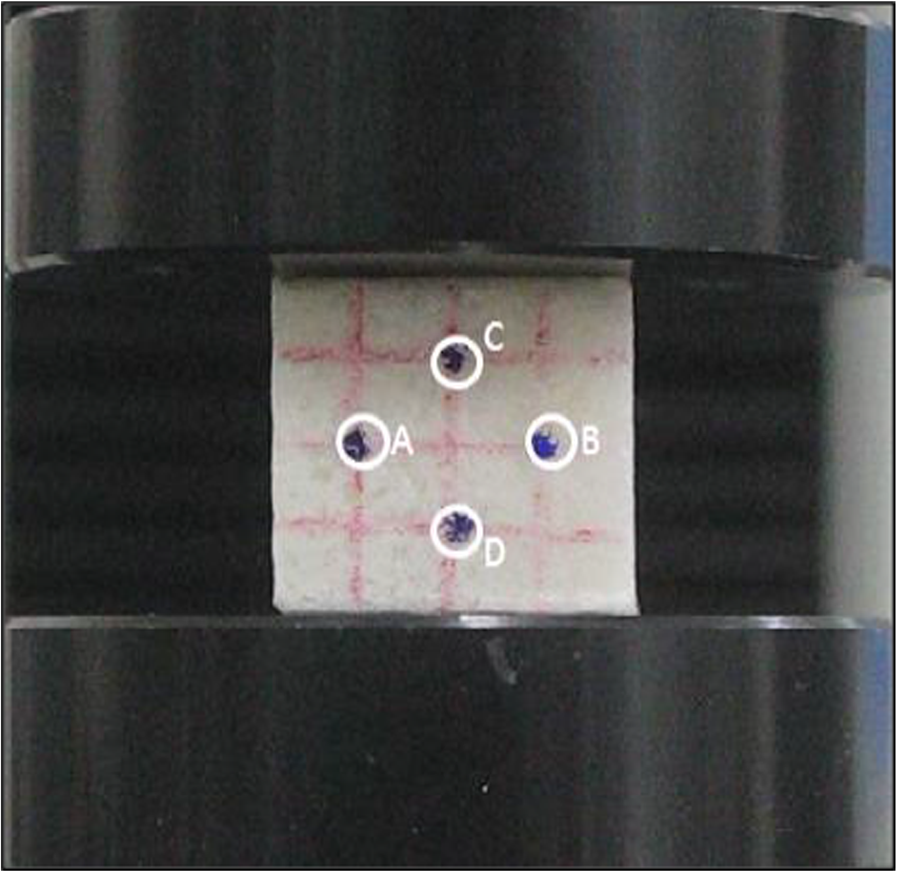
Marks for estimating Poisson’s ratio of specimens. Initial points. **a**–**d** (before compressive pressure)

**Fig. 2:**
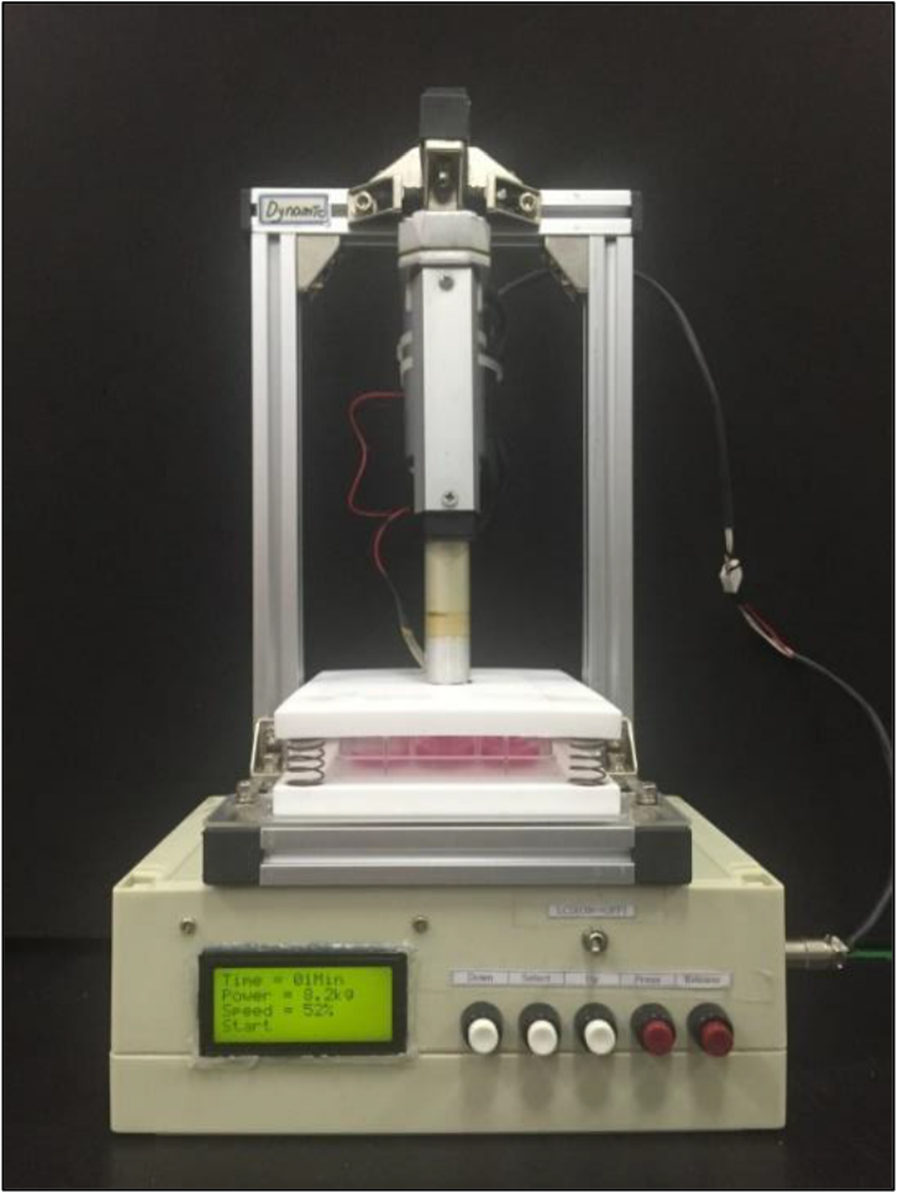
An apparatus for cell culture with compressive stimulation. The apparatus was manufactured to apply 10 % compressive strain to the scaffold in 12-well plate

To determine the Poisson’s ratio, scanning electron microscope (SEM) images of scaffolds were analyzed using Image J V2.0.0. The Material Testing System (LRX-PLUS, Lloyd Instruments, West Sussex, UK) was utilized to apply a load to the specimens. A digital microscope (BX51, Olympus Corporation, Tokyo, Japan) was used to capture images and measure the displacement of the specimen [Fig. 1]. All specimens were compressed from 0 % to 25 % strain and the image was captured with 5 % strain intervals. The relative positions of pointed marks were used to estimate strain and Poisson’s ratio of the specimens. Points A–D was used to estimate the Poisson’s ratio by tracking the center of the points, as shown in Fig. 1. The changes in the x-axis and y-axis distance were calculated by Equations (1) and (2), and these values were input into Equation (3) to determine the Poisson’s ratio [18, 19] [Fig. 2].

1 $$ {\upvarepsilon}_{\mathrm{x}} = \frac{\left|A-B\right|-\left|A0-B0\right|\ }{\left|A0-B0\right|} $$

2 $$ {\upvarepsilon}_{\mathrm{y}} = \frac{\left|C-D\right|-\left|C0-D0\right|\ }{\left|C0-D0\right|} $$

3 $$ \nu = - \frac{\upvarepsilon \mathrm{x}}{\ \upvarepsilon \mathrm{y}} $$

where: ε_x_ = strain of x-axis

ε_y_ = strain of y-axis

ν = material Poisson’s ratio

A0 ~ D0 = initial point (load = 0)

A ~ D = moved point under load

### Measurement of contact angle

The polymer film was fabricated by solvent evaporation methods to measure surface properties of PLGA and HA/PLGA composite. Contact angle of HA/PLGA composite was analyzed according to the HA content in the PLGA using a contact angle analyzer (Phoenix 250, Surface & Electro-Optics Corporation, Korea) and an analysis program (Image Pro 300, Surface & Electro-Optics Corporation, Korea).

### Mechanical properties on wet/dry state of the specimens

Compressive strength of the scaffold was estimated in wet state for simulating cell culture environment using a Material Testing System (MTS) at 10 % compressive strain. The compressive strength of the composite scaffold was examined at 10 and 15 % strain. The cross head speed of MTS was 1 mm/min. Both HA/PLGA composite and PLGA-only scaffolds were pre-wetted with ethanol for 2 h and then submerged in PBS solution at 37 °C for 2 h. After pre-wetting the specimens, they were soaked in DMEM at 37 °C for 2 h in shaking incubator at 200 rpm [15]. Measurement of the stress–strain recovery rate of the scaffold was conducted after applying 10 and 15 % compressive strain for 5 min to the scaffold, then, recovered position was measured after 5 min from releasing load. The recovery rate was measured at a ratio to return to its original position after applying compression to the specimens. The recovery rate was obtained according to Equation (4).

4 $$ \mathrm{Recovery}\ \mathrm{rate} = \frac{A\  recovery\  length(mm)}{Applying\ a\  compression\  length(mm)} $$

### Cell culture and proliferation with dynamic compressive stimulation

Human osteoblastic-like cells (MG-63, KCLB, Seoul, Korea) were cultured in cell culture flasks with DMEM supplemented with 10 % FBS and 1 % P/S in a humidified incubator with 5 % CO2 at 37 °C. The media was exchanged every 3 days. For sterilization, the specimens were incubated in 70 % ethanol for 1 h, followed by washing three times with PBS solution. For all cell tests, 4 to 6 passaged MG-63 cells were used. For cell proliferation test, cells were seeded on the scaffolds at an initial cell density of 2.0 × 105 (cells/specimen) and cultivated in a humidified incubator containing 5 % CO2 at 37 °C. Dynamic compressive stimulation was carried out for 4 h per a day and consisted of 0-, 5-, and 15-min cycle of 15 % compressive strain (approximately 19.6 N).

The cell proliferation rate was measured using a Cell Counting Kit (CCK-8, Dojindo Molecular Technologies, Inc., Maryland, USA). The cell proliferation rates were observed on days 1, 3, and 5. CCK-8 was mixed with scaffold’s medium (DMEM:CCK solution = 10:1) at each days of 1, 3, and, 5, and then placed in an incubator at 37°C for 4 h. Finally, the 1, 3, and 5 days’ absorbance of the CCK-8 solution was measured using a Fluorescence Multi-Detection Reader (Synergy HT, BIO-TEK Instruments, Inc., Vermont, USA) at 450 nm.

### Statistical analysis

Data were expressed as the means ± standard error for all comparisons. A **t**-test was used to evaluate differences between groups (**p <**0.05).

## Results and Discussion

### Mechanical properties of prepared scaffolds

**Fig. 3:**
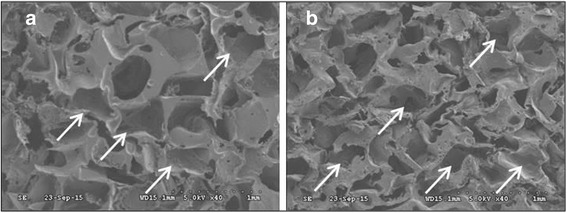
Cross-sectional SEM images of (**a**) PLGA, and (**b**) 10 wt. % HA/PLGA composite scaffolds after tri-axial compression. The white arrow marks indicate different pore structures (scale bar = 1 mm)

NPR was successfully achieved in the HA/PLGA composite scaffold. NPR resulted from transformation of microstructural shapes of the scaffolds. The morphology of pores was transformed from convex to concave shape, which enables the ratios property in the scaffolds as shown in Fig. 3. Both PLGA and HA/PLGA composite scaffolds contained concave and dented pores as indicated by the white arrow marks in Fig. 3(a) and (b). These recessed shapes could be deemed as having typical pore structures for a negative Poisson’s ratio.

**Fig. 4:**
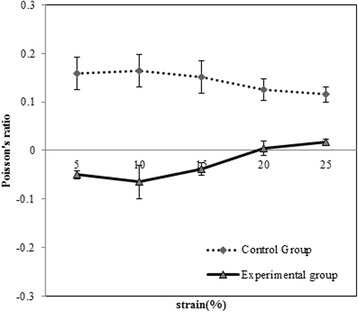
Poisson’s ratio of PLGA scaffolds under 5 ~ 25 % strain with compressive loading. (**n =** 5)

Poisson’s ratio of conventional (control) scaffold specimens were 0.15 ~ 0.24 range when compressed at 5 ~ 25 % strain. However, the NPR (experimental) scaffold specimens were shown negative value as −0.01 ~ −0.07 range at 5 ~ 25 % compressive strain. The lowest Poisson’s ratio was −0.07 at 10 % strain level with compression at NPR (experimental) scaffold. (Fig. 4)

In this study, we investigate Poisson’s rstio of the scaffolds in dry state. We thought that it is nessessary to investigate the behavior of the scaffolds in wet state. In next stduty, we will investigate Poisson’s ratio of the scaffolds in wet state.

**Fig. 5:**
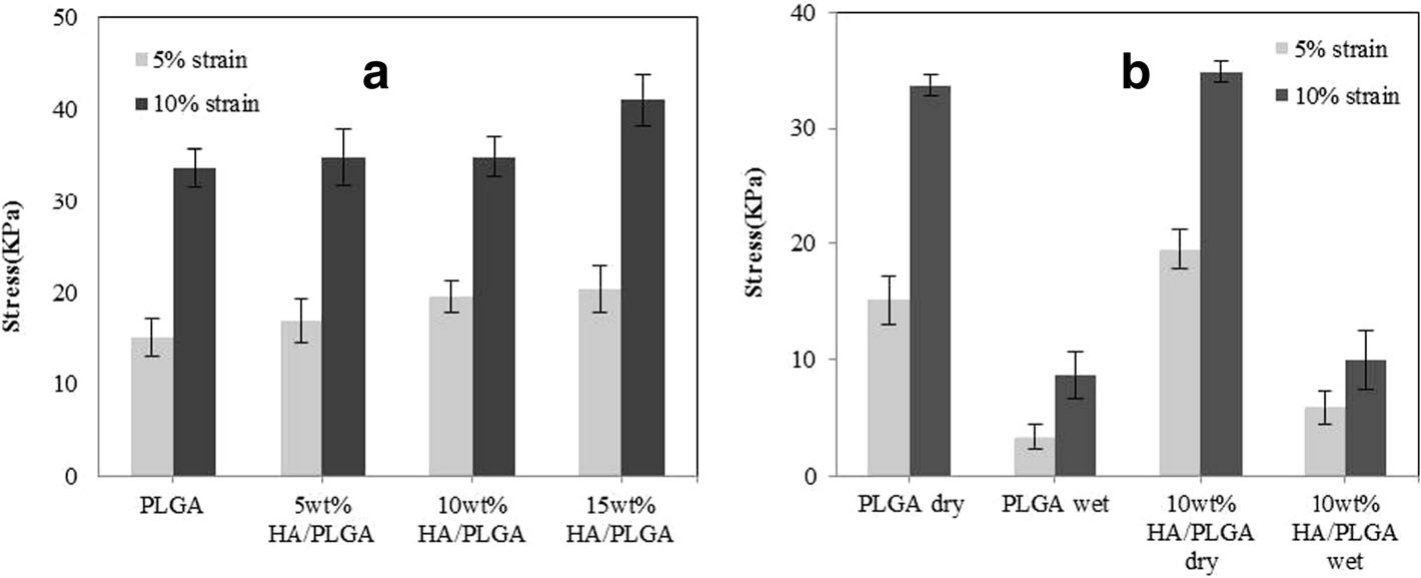
Mechanical property of each scaffold specimen at 5 and 10 % strain. **a** various HA contents and (**b**) in wet and dry state (**n =** 4, *p < 0.05)

The mechanical properties of the scaffolds were shown in Fig. 5(a). The compressive strength of the scaffolds was increased by incorporation of HA. Mechanical strength of 15 wt. % HA/PLGA composite scaffold was 21.7 % higher than that of PLGA scaffold. Mechanical properties of the scaffolds in dry/wet state at 5 % and 10 % strain (compression) were shown in Fig. 5 (b).

The mechanical strength of the PLGA scaffold tended to decrease dramatically in wet state, with an overall reduction of about 70 % compared to those in dry state. However, that of the composite scaffolds increased in response to the addition of HA in dry and wet state. Therefore, HA incorporated composite PLGA scaffold was increased in compressive strength and then delivered physical stimulation to the cells in wet condition.

**Fig. 6:**
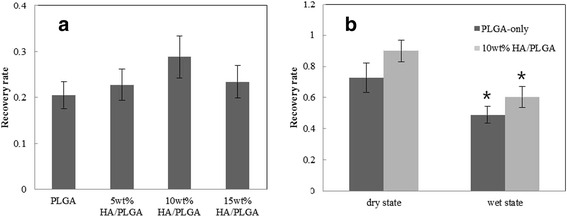
Effect of wet/dry state with HA content on recovery rate (**a**) 10 % strain on wet state and (**b**) 15 % strain on dry and wet state (**n** *=* 4, *p < 0.05)

In wet state, the scaffolds could not recover to original shape after compression due to loss of elasticity. The mechanical property of the scaffold is important to transfer physical stimulation to cells during cultivation in culture media. So, the recovery rate of scaffold was investigated to determine the endurance of scaffold under dynamic loading stimulation during cell cultivation. The recovery rates of scaffolds in wet and dry state at 10 and 15 % strain level are shown in Fig. 6(a) and (b), respectively. The recovery rate of the PLGA scaffold was increased with increasing HA contents under dry and wet state. In 10 % strain compression, the recovery rate of 10 wt. % HA/PLGA composite scaffold was increased up to 29 % of compared with PLGA-only of 20 %, however, the recovery rate of 15 wt. % HA/PLGA composite scaffold was decreased about 23 % compared with 10 wt. % HA/PLGA composite scaffold [Fig. 6 (a)]. In 15 % strain compression, the recovery rate of 10 wt. % HA/PLGA composite scaffold was 23.4 % higher in dry-state and 22.4 % higher in wet-state than PLGA-only scaffold [Fig. 6 (b)]. It was shown 10 wt. % HA/PLGA composite scaffold’s recovery rate is highest compared to PLGA-only scaffold. Therefore, 10 wt. % HA/PLGA composite scaffold would be better to deliver dynamic mechanical stimuli effectively to the cells than PLGA-only scaffold in wet condition.

**Fig. 7:**
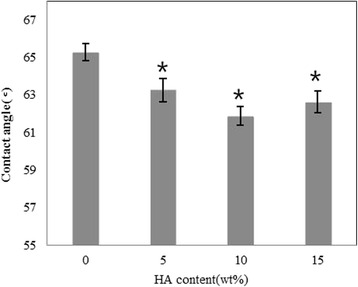
Contact angles according to Hydroxyapatite contents (*n =* 4, *p < 0.05)

Contact angle of the HA/PLGA composite scaffold was measured to determine hydrophilicity of the specimens. Hydrophilicity of the composite specimen films was increased with increasing HA content [Fig. 7]. However, when the amount of HA content exceeded 10 %, contact angle of the composite films gradually increased and showed relatively hydrophobic property. These findings indicated that an optimized content (10 %) of HA is necessary to maintain hydrophilicity of the PLGA scaffold.

### Cell culture and proliferation with dynamic compressive stimulation

**Fig. 8:**
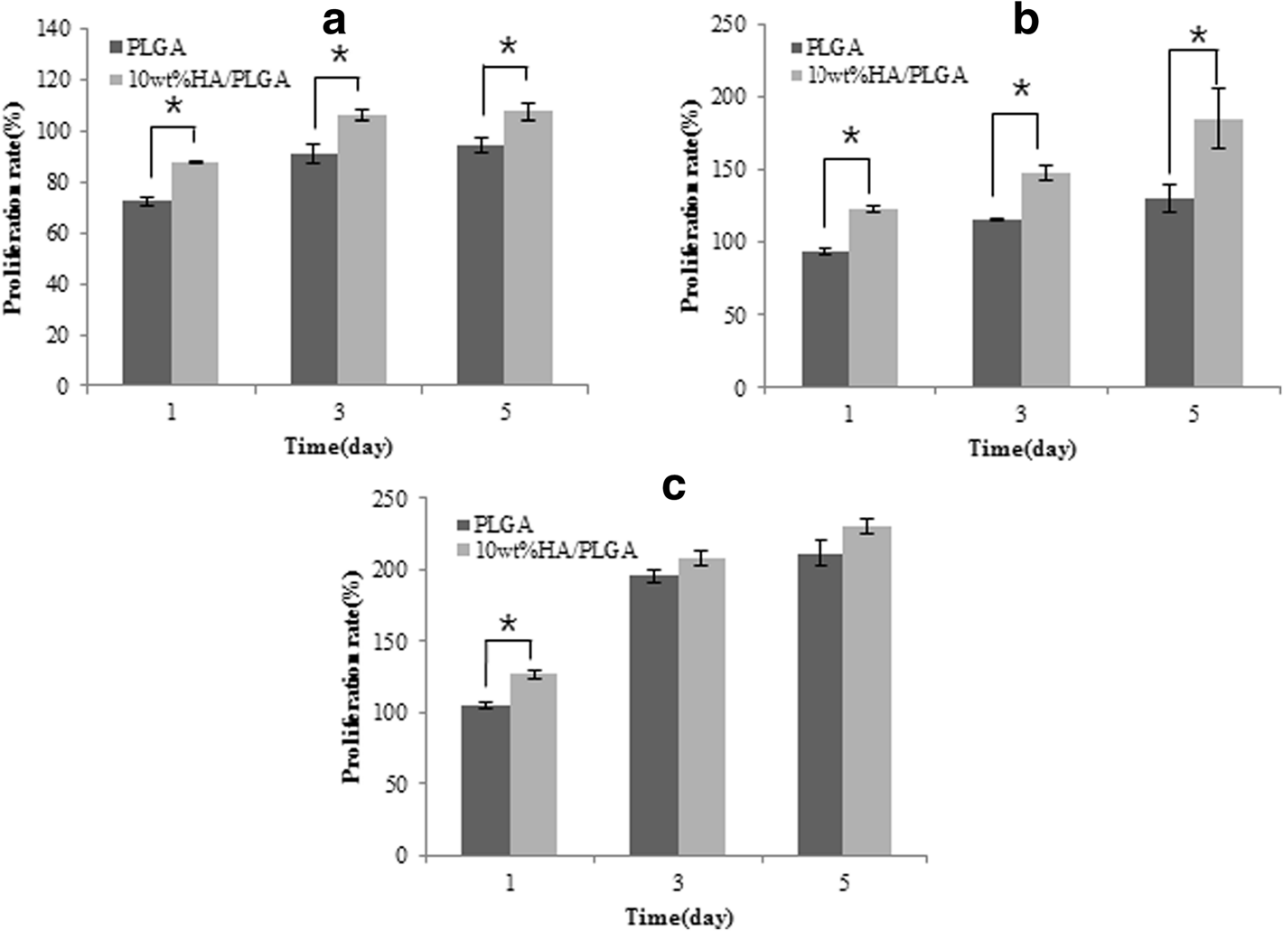
MG-63 cell proliferation rate on PLGA and HA/PLGA scaffolds (**a**) without dynamic compressive stimulation, (**b**) with dynamic compression stimulation (5 min cycles), (**c**) with dynamic compression stimulation (15 min cycles) at 1, 3 and 5 days of culture (*n =* 4, *p < 0.05)

In cell proliferation tests, proliferation of the MG-63 cells in the HA/PLGA composite scaffold without dynamic compression stimulation was 15–20 % higher than that of PLGA-only scaffold at 5 days of cultivation [Fig. 8 (a)]. These findings indicated that HA incorporation may facilitate bone cell growth. Considering that human bone is composed of 69 % HA, the addition of the calcium phosphate-based ceramic HA likely provided the most similar properties to natural bone.

**Fig. 9:**
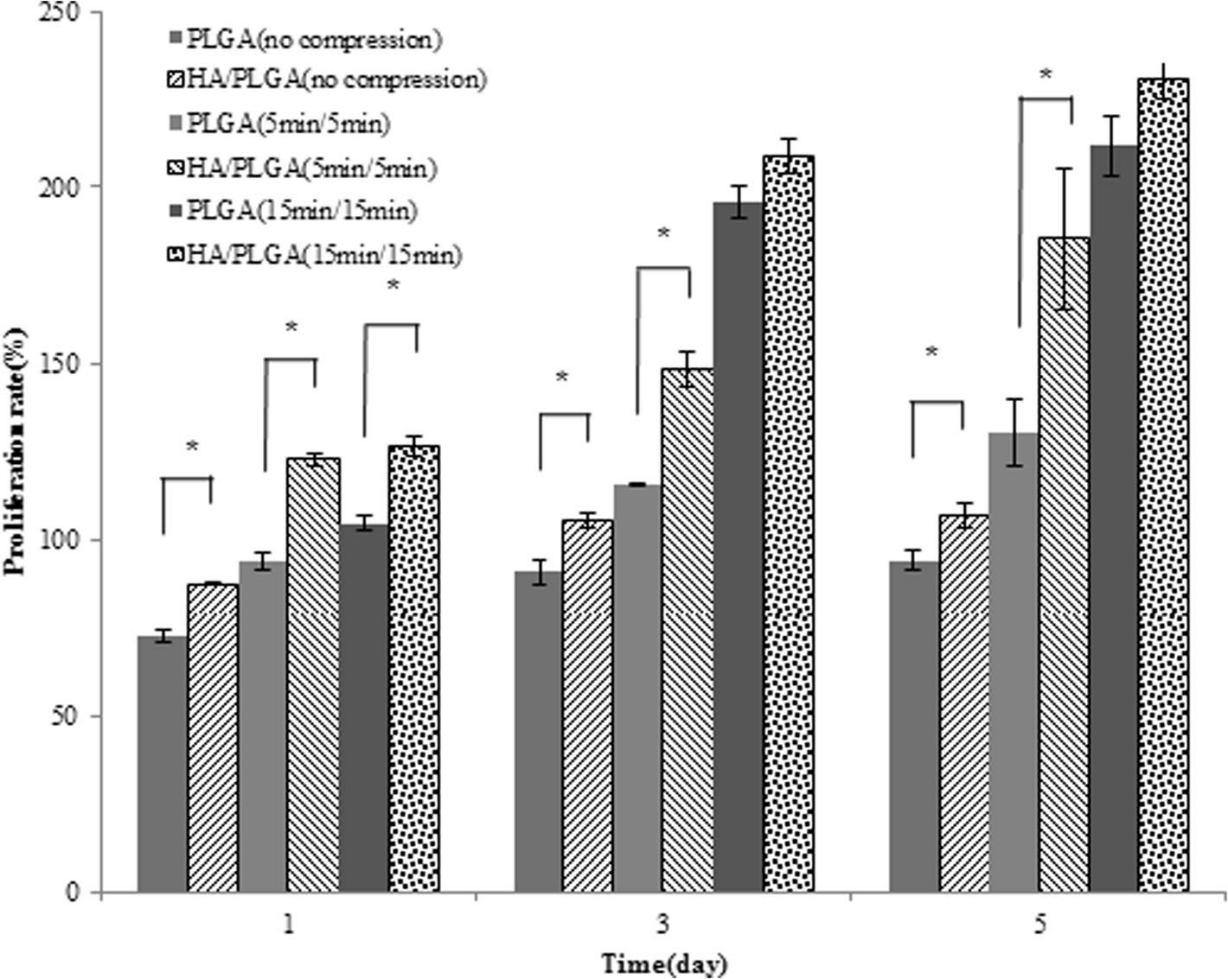
MG-63 cell proliferation rate on PLGA and HA/PLGA scaffolds with various dynamic compression at 1, 3 and 5 days of culture (*n =* 4, *p < 0.05)

In cell culture with 5 min-cycle intervals of compressive stimulation, MG-63 cell proliferation was increased about 30.6, 28.4 and 42.1 % higher in 10 wt. % HA/PLGA composite scaffold compared with PLGA-only scaffold for 1, 3 and 5 days culture, respectively [Fig. 8 (b)]. The result of cell proliferation at 5 min-cycle stimuli showed a statistically significant difference. On the other hand, in case of cells proliferation with 15 min-cycle stimulation, 10 wt. % HA/PLGA composite scaffolds showed higher cell proliferation than PLGA-only scaffolds, but did not show statistically significant difference at 3 and 5 days culture [Fig. 8 (c), Fig. 9].

Our comprehensive analysis of cell proliferation test showed that just addition of HA, a major component of bone, increased the cell proliferation of PLGA scaffold. Furthermore, applying suitable stimulation-cycle (this study was 5 min-cycle) further increased bone-cell proliferation in the scaffold. Therefore, more frequent stimulating during a short-cycle at NPR composite scaffold provides effective isotropic compression for bone cell proliferation.

## Conclusions

We successfully fabricated the HA/PLGA composite scaffold having a negative Poisson’s ratio. We found a suitable HA content that was improved the hydrophilicity and mechanical property of the PLGA scaffold. The addition of HA increased the compression strength of the PLGA scaffold in the wet-state and positively contributed to stimuli-cell culture environment. The HA/PLGA composite scaffold can maintain their shape during the continuous compressive stimulation.

In case of without compression stimulation, the addition of 10 wt. % HA led to 20 % increases in osteoblast-like cell proliferation relative to PLGA at 5 days of culture. We also confirmed that 10 wt. % HA/PLGA composite scaffold showed 2 times higher cell proliferation than PLGA scaffold following dynamic stimulation. Hence, proper dynamic compression stimulation (5 min cycles) would facilitate bone regeneration by supplying an effective isotropic compression in NPR scaffold.

Overall, these findings indicate that the NPR composite scaffolds used in this study would help bone regeneration by supplying a better osteo-compatibility and an effective isotropic compression to stimulate the proliferation of bone cells.

## Abbreviations

CCK-8: Cell, counting kit-8
DMEM: Dulbecco’s modified Eagle’s medium
MTS: Material Testing System
NPR: Negative Poisson ratio
PBS: Phosphate buffered saline solution
PLGA: Poly (D, L-lactic-co-glycolic acid)
SEM: Scanning electron microscope
